# Long-Term l-Serine Administration Reduces Food Intake and Improves Oxidative Stress and Sirt1/NFκB Signaling in the Hypothalamus of Aging Mice

**DOI:** 10.3389/fendo.2018.00476

**Published:** 2018-08-23

**Authors:** Xihong Zhou, Haiwen Zhang, Liuqin He, Xin Wu, Yulong Yin

**Affiliations:** ^1^Key Laboratory of Agro-Ecological Processes in Subtropical Region, Institute of Subtropical Agriculture, Chinese Academy of Sciences, Changsha, China; ^2^Key Laboratory of Tropical Animal Breeding and Epidemic Disease Research of Hainan Province, Hainan University, Haikou, China; ^3^Laboratory of Animal Nutrition and Human Health, College of Life Sciences, Hunan Normal University, Changsha, China

**Keywords:** aging, inflammation, oxidative stress, serine, Sirt1

## Abstract

Serine has recently been shown to reduce oxidative stress and inflammation, which, when occurring in the hypothalamus, contribute to age-related obesity. To explore whether long-term serine administration reduces oxidative stress and body weight in aging mice, various concentrations of l-serine dissolved in water were administered to 18-month-old C57BL/6J mice for 6 months. The results showed that the administration of 0.5% (w/v) l-serine significantly reduced food intake and body weight gain during the experiment. Moreover, the administration of 0.5% l-serine decreased the concentrations of leptin, malondialdehyde, interleukin-1β, and interleukin-6, while it increased those of superoxide dismutase and glutathione, in both the serum and hypothalamus. Reactive oxygen species and the activity of nicotinamide adenine dinucleotide phosphate oxidase were reduced in the hypothalamus of aging mice treated with l-serine as compared with untreated control mice. Additionally, the expression of the leptin receptor increased while the levels of neuropeptide Y and agouti-related protein decreased in mice that had been treated with 0.5% l-serine. The expression of Sirt1 and phosphorylated signal transducers and activators of transcription 3 (pSTAT3) increased, while that of phosphorylated NFκB decreased in the mice treated with 0.5% l-serine. These results indicated that long-term l-serine administration reduces body weight by decreasing orexigenic peptide expression and reduces oxidative stress and inflammation during aging in mice, possibly by modulating the Sirt1/NFκB pathway. Thus, l-serine has the potential to be used in the prevention of age-related obesity.

## Introduction

The hypothalamus is a critical part of the central nervous system that modulates the stress response and senses nutrient-related inputs. However, in aging mice, increased levels of oxidative stress and inflammatory markers are observed in the hypothalamus (1–3). Low levels of antioxidant enzymes and high levels of lipid peroxidation make the hypothalamus vulnerable to reactive oxygen species (ROS) (4–6). These elevations of oxidative damage and inflammatory responses in the central nervous system are believed to contribute to age-related diseases including obesity and various neurodegenerative disorders (7–9).

The nicotinamide adenine dinucleotide-dependent deacetylase sirtuin 1 (Sirt1) regulates the response to oxidative stress, which correlates with many diseases (10–12). Sirt1 activity in the hypothalamus decreases in association with aging, and its low expression contributes to a low level of antioxidants and increased oxidative damage (10). Importantly, Sirt1 is also a nutrient sensor and plays critical roles in the energy balance in the hypothalamus. Debate is ongoing as to whether hypothalamic Sirt1 has orexigenic or anorexigenic effects, although most of the evidence shows that inhibition of Sirt1 induces a negative energy balance by regulating the activity of forkhead transcription factor FKHR (FOXO1) (13–15) and the expression of agouti-related protein (AGRP) and neuropeptide Y (NPY) (16, 17). Additionally, the transcription factor nuclear factor-κB (NFκB) regulates numerous target genes to exert its biological functions. Importantly, NFκB functions as a central regulator of the immune response and controls the secretion of inflammatory cytokines in many tissues including the hypothalamus (18–20). Moreover, NFκB is also a central mediator of stress responses under conditions of oxidative stress and upon exposure to certain chemicals in the central nervous system (18).

l-Serine is traditionally considered a non-essential amino acid. Recently, dietary supplementation with l-serine has been shown to have antioxidant effects (21–24). l-Serine is a precursor of glycine and cysteine, which can be used for the synthesis of glutathione. In addition, l-serine exerts critical functions in the mammalian central nervous system. It serves as a precursor of the neuroactive substances l-serine and glycine, and mediates neuroprotective effects (25, 26). However, it remains unknown whether l-serine has any effects on age-related oxidative stress in the hypothalamus. Consequently, we conducted the present study to explore the effects of long-term l-serine supplementation on hypothalamic oxidative stress and Sirt1 expression in aging mice.

## Materials and methods

### Animals

Eighty 18-month-old C57BL/6J male mice were included in the study. The mice were obtained from SLAC Laboratory Animal Center (Changsha, China) and were housed in a temperature-controlled animal facility (lighting cycle: 12 h/d), with free access to food and water. All animals were randomly assigned into four groups: control mice, which were kept untreated, and mice administered with 0.1, 0.2, or 0.5% (w/v) l-serine dissolved in the drinking water. Eighteen-month-old adult mice were used as young controls. The experiment had the duration of 6 months. Food intake and body weight were recorded every week. Upon completion of the experiment, mice were euthanized with isoflurane and the blood was collected from the retro-orbital sinus. The hypothalamus was excised and collected according to the method described in a previous study (7). The hypothalamus samples were either immediately snap-frozen in liquid nitrogen or fixed in formaldehyde solution. This study was performed in accordance with the recommendations of the Guide for Care and Use of Laboratory Animals published by the Animal Welfare Committee of the Institute of Subtropical Agriculture, Chinese Academy of Sciences. The protocol was approved by the Animal Welfare Committee of the Institute of Subtropical Agriculture, Chinese Academy of Sciences.

### Biochemical assays

Superoxide dismutase (SOD), malondialdehyde (MDA), and glutathione (GSH) biochemical assays were performed with commercially available kits from Nanjing Jiancheng Bioengineering Institute (Nanjing, Jiangsu, China). Interleukin (IL)-1β, IL-6, and leptin biochemical assays were performed with kits from Cusabio Biotech (Wuhan, Hubei, China; https://www.cusabio.com/). Nicotinamide adenine dinucleotide phosphate (NADPH) oxidase activity was assayed as previously reported (27).

### Determination of reactive oxygen species

ROS content was determined as previously described (21). Hypothalamus samples were placed in optimum cutting temperature compound (Sakura Finetek, Tokyo, Japan) and flash-frozen in a methylbutane-chilled bath at −81 ± 2°C, were sliced into 10-μm sections and stained with a solution of 1 μM dihydroethidium (Sigma-Aldrich, St. Louis, MO, USA), for 20 min, at 37°C in a humidified 5% CO_2_ incubator. Results were observed and analyzed by fluorescence microscopy and Image Browser software (Leica, Wetzlar, Germany).

### Immunohistochemistry assay

Hypothalamus samples were cut into 4-μm sections and processed for immunohistochemical staining as previously described. Briefly, the samples were incubated with a primary antibody [leptin receptor (LepRb), NPY, or AGRP; Boster, Wuhan, China] overnight at 4°C and then with poly-horseradish peroxidase-conjugated IgG for 60 min at 22 ± 4°C. Subsequently, the avidin-biotin-peroxidase complex and the substrate 3,3′-diaminobenzidine were applied for 2 min and the samples were analyzed.

### RT-qPCR assay

Total hypothalamic RNA was isolated using the TRIzol® reagent and reverse-transcribed into cDNA with the PrimeScript™ RT reagent kit (TaKaRa Bio, Otsu, Japan). Quantitative PCR was performed as previously reported (28). Briefly, the reaction was performed with a total volume of 10 μL assay solution containing 5 μL SYBR® Green mix (TaKaRa Bio), 0.2 μL ROX internal reference dye, 1 μL cDNA template, 3 μL deionized H_2_O, and 0.4 μL each of the forward and reverse primers. The comparative Ct method was applied to calculate the mRNA expression of the target genes relative to that of β-actin. The primer sequences are presented in Supplementary Table 1.

### Western blot assay

Protein supernatants extracted from hypothalamus samples were run on 10% sodium dodecyl sulfate acrylamide gels and electro-blotted onto nitrocellulose membranes. The membranes were incubated with primary antibodies overnight at 4°C, followed by a second incubation with anti-rabbit or anti-mouse IgG. Primary antibodies specific for Sirt1, STAT3, phospho-STAT3, and total NFκB (Cell Signaling Technology, Beverly, MA, USA) were used. Finally, the EZ-ECL chemiluminescence reagent (Biological Industries, Cromwell, CT, USA) was added onto the membrane and bands were obtained.

### Statistical analysis

All statistical analyses were performed with one-way analysis of variance, using the general linear model and the MIXED procedure (PROC MIXED) from SAS software version 9.2 (SAS Institute Inc., Cary, NC, USA). Data are presented as least squares means ± standard error of the mean. Mean values were considered significantly different when *P* < 0.05.

## Results

### Effects of long-term l-Serine administration on body weight and food intake in aging mice

**Figure 1:**
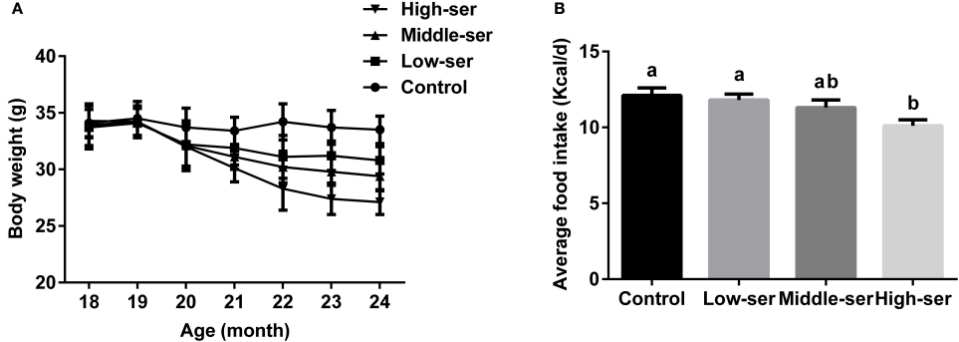
Effects of long-term l-serine administration on body weight and food intake in aging mice. **(A)** Body weight; **(B)** average daily food intake. Control, untreated control mice; Low-ser, mice supplemented with 0.1% (wt/vol) l-serine dissolved in the drinking water; Middle-ser, mice supplemented with 0.2% (wt/vol) l-serine dissolved in the drinking water; High-ser, mice supplemented with 0.5% (wt/vol) l-serine dissolved in the drinking water. Values are expressed as mean ± standard error of the mean (SEM), *n* = 20; ^a, b^Means of the bars with different letters were significantly different among groups (*P* < 0.05).

The long-term administration of 0.5% l-serine significantly decreased body weight and reduced food intake in aging mice when compared with controls (i.e., aging mice that did not receive l-serine), while the administration of 0.1 or 0.2% l-serine did not show such effects (Figure 1).

### Effects of long-term l-Serine administration on oxidative stress in aging mice

**Figure 2:**
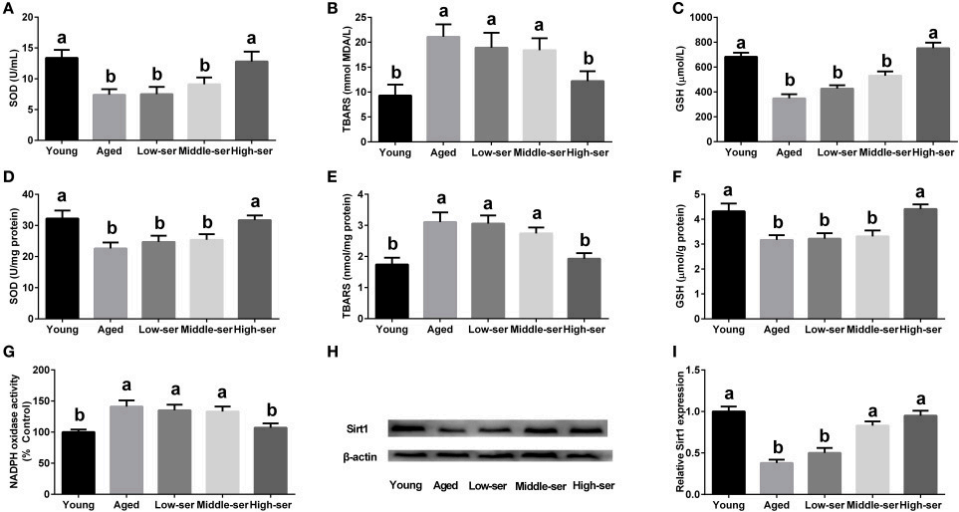
Effects of long-term l-serine administration on oxidative stress in aging mice. **(A–C)** SOD, MDA, and GSH contents in serum; **(D–F)** SOD, MDA, and GSH contents in the hypothalamus; **(G)** NADPH oxidase activity; **(H**,**I)** Protein expression of Sirt1. Aged, untreated control mice; Low-ser, mice supplemented with 0.1% (wt/vol) l-serine dissolved in the drinking water; Middle-ser, mice supplemented with 0.2% (wt/vol) l-serine dissolved in the drinking water; High-ser, mice supplemented with 0.5% (wt/vol) l-serine dissolved in the drinking water. Young, adult male mice at the age of 18 months. SOD, superoxide dismutase; MDA, malondialdehyde; GSH, glutathione; NADPH, nicotinamide adenine dinucleotide phosphate. Values are expressed as mean ± SEM, *n* = 3 for the statistical analysis of western blotting data and *n* = 8 for the statistical analysis of other data; ^a, b^Means of the bars with different letters were significantly different among groups (*P* < 0.05).

**Figure 3:**
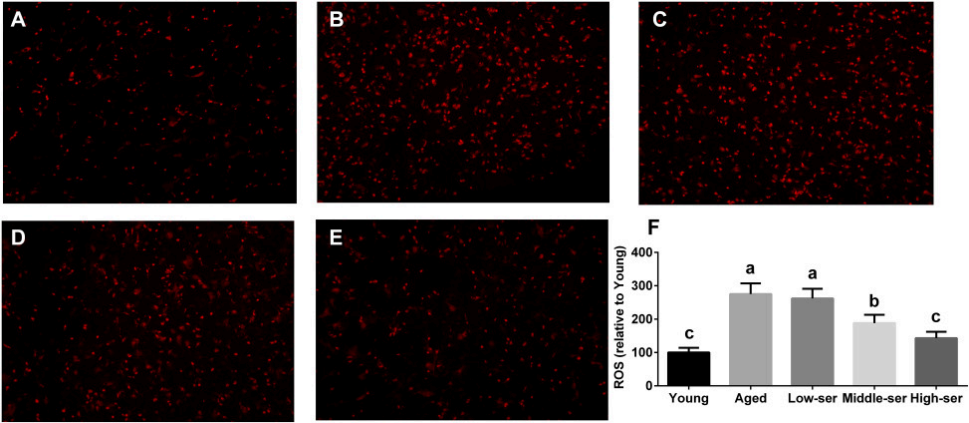
Effects of long-term l-serine administration on the ROS content in aging mice. Representative images of ROS in mice from the Young **(A)**, Aged **(B)**, Low-ser **(C)**, Middle-ser **(D)**, and High-ser **(E)** groups; **(F)** Relative ROS content. Aged, untreated control mice; Low-ser, mice supplemented with 0.1% (wt/vol) l-serine dissolved in the drinking water; Middle-ser, mice supplemented with 0.2% (wt/vol) l-serine dissolved in the drinking water; High-ser, mice supplemented with 0.5% (wt/vol) l-serine dissolved in the drinking water. Young, adult male mice at the age of 18 months. ROS, reactive oxygen species. Values are expressed as mean ± SEM, *n* = 4. ^a, b, c^Means of the bars with different letters were significantly different among groups (*P* < 0.05).

The long-term administration of 0.5% l-serine significantly increased the SOD and GSH levels while decreasing the MDA contents in both the serum and hypothalamus of aging mice in comparison with controls (Figures 2A–F). However, the administration of l-serine at 0.1 or 0.2% had no effect. Additionally, the administration of 0.5% l-serine significantly decreased NADPH oxidase activity (Figure 2G) and ROS content (Figure 3) while it increased Sirt1 expression (Figures 2H,I) in the hypothalamus.

### Effects of long-term l-Serine administration on the inflammatory response in aging mice

**Figure 4:**
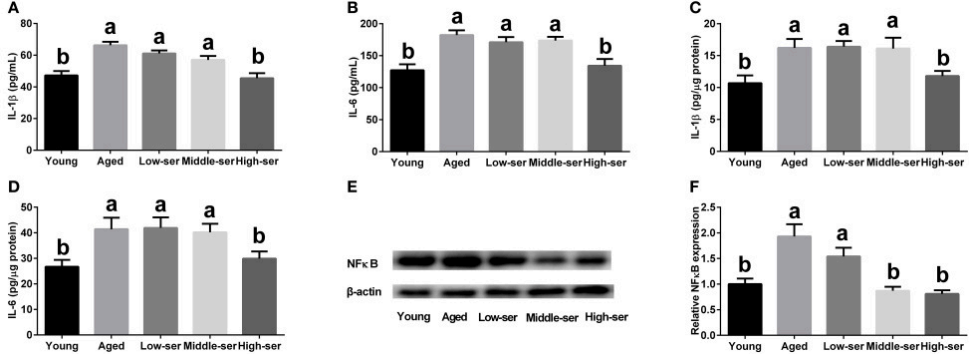
Effects of long-term l-serine administration on the inflammatory response in aging mice. **(A,B)** Concentrations of IL-1β and IL-6 in serum; **(C,D)** Concentrations of IL-1β and IL-6 in the hypothalamus; **(E,F)** Relative NF-κB protein expression. Aged, untreated control mice; Low-ser, mice supplemented with 0.1% (wt/vol) l-serine dissolved in the drinking water; Middle-ser, mice supplemented with 0.2% (wt/vol) l-serine dissolved in the drinking water; High-ser, mice supplemented with 0.5% (wt/vol) l-serine dissolved in the drinking water. Young, adult male mice at the age of 18 months. Values are expressed as mean ± SEM, *n* = 3 for the statistical analysis of western blotting data and *n* = 8 for the statistical analysis of other data; ^a, b^Means of the bars with different letters were significantly different among groups (*P* < 0.05).

The long-term administration of 0.5% l-serine significantly decreased the concentrations of IL-1β and IL-6 in the serum and hypothalamus of aging mice when compared with the control animals. By contrast, l-serine at 0.1 or 0.2% had no effect (Figures 4A–D). In addition, the long-term administration of 0.5% l-serine significantly decreased NFκB expression in the hypothalamus (Figures 4E,F).

### Effects of long-term l-Serine administration on orexigenic neuropeptide expression in aging mice

**Figure 5:**
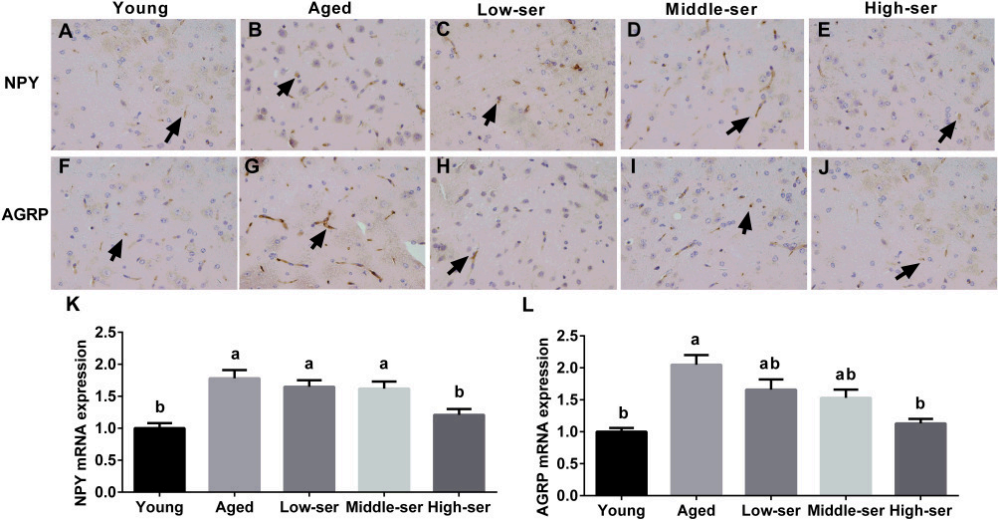
Effects of long-term l-serine administration on orexigenic neuropeptide expression in aging mice. NPY **(A–E)** and AGRP **(F–J)** expression as determined by immunohistochemistry; relative NPY **(K)** and AGRP **(L)** mRNA expression. Aged, untreated control mice; Low-ser, mice supplemented with 0.1% (wt/vol) l-serine dissolved in the drinking water; Middle-ser, mice supplemented with 0.2% (wt/vol) l-serine dissolved in the drinking water; High-ser, mice supplemented with 0.5% (wt/vol) l-serine dissolved in the drinking water. Young, adult male mice at the age of 18 months. Arrow (yellow), expression of target proteins; NPY, neuropeptide Y; AGRP, agouti-related protein. Values are expressed as mean ± SEM, *n* = 8; ^a, b^Means of the bars with different letters were significantly different among groups (*P* < 0.05).

Long-term administration of 0.5% l-serine significantly decreased NPY and AGRP expression in the hypothalamus of aging mice in comparison with controls; however, administration of L-serine at 0.1 or 0.2% showed no such effects (Figure 5).

### Effects of long-term l-Serine administration on leptin signal pathway in aging mice

**Figure 6:**
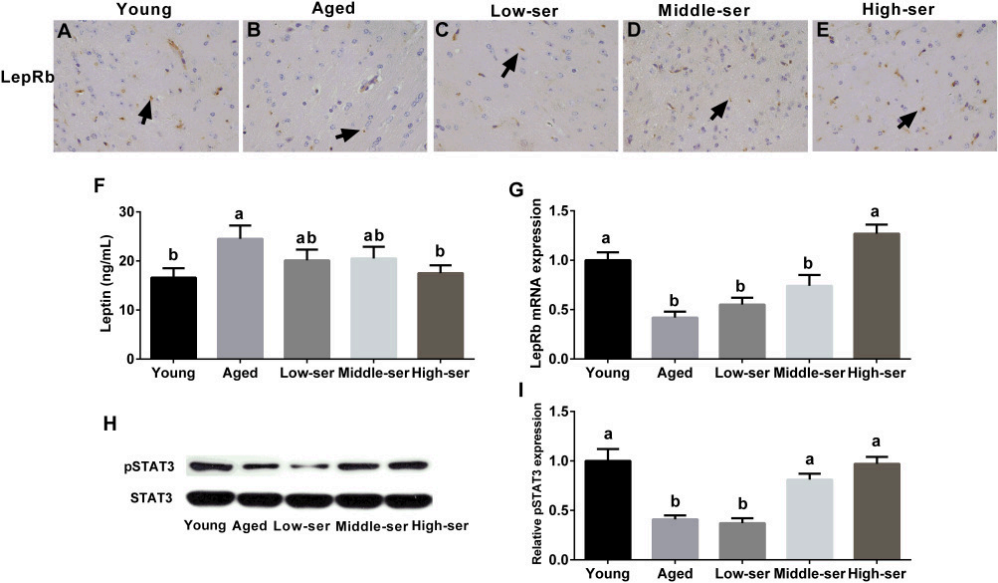
Effects of long-term l-serine administration on the leptin signaling pathway in aging mice. **(A–E)** LepRb expression as determined by immunohistochemistry; **(F)** leptin content in the hypothalamus; **(G)** relative LepRb mRNA expression; **(H,I)** relative pSTAT3 protein expression. Aged, untreated control mice; Low-ser, mice fed with 0.1% (wt/vol) l-serine dissolved in the drinking water; Middle-ser, mice fed with 0.2% (wt/vol) l-serine dissolved in the drinking water; High-ser, mice fed with 0.5% (wt/vol) l-serine dissolved in the drinking water. Young, adult male mice at the age of 18 months. Arrow (yellow), expression of LepRb protein; LepRb, leptin receptor. Values are expressed as mean ± SEM, *n* = 3 for the statistical analysis of western blotting data and *n* = 8 for the statistical analysis of other data; ^a, b^Means of the bars with different letters were significantly different among groups (*p* < 0.05).

The long-term administration of 0.5% l-serine, but not 0.1 or 0.2% l-serine, significantly decreased the leptin content (Figure 6F) in the serum and increased LepRb expression (Figures 6A–E,G) in the hypothalamus of aging mice when compared with controls. In addition, the long-term administration of 0.5% l-serine significantly increased the expression of phosphorylated STAT3 in the hypothalamus of aging mice (Figures 6H,I).

## Discussion

Our previous studies have shown that l-serine could alleviate oxidative stress in various tissues of different rodent models, including diquat- and long-term high-fat-induced oxidative stress in liver (21, 22) and lipopolysaccharides-induced oxidative damage in the small intestine (23). Based on these previous experiments, we chose the dose of 0.1, 0.2, and 0.5% (w/v) l-serine for the current 6-month study, respectively. Additionally, since patients who received the relatively high amount of 30 g/day l-serine for 9 months in phase II clinical trials did not show any side effects and l-serine is generally regarded as safe by the United States Food and Drug Administration (25), we employed l-serine as a non-toxic agent. As expected, our results further supported that long-term l-serine supplementation may alleviate age-related oxidative damage, since it is shown herein that l-serine increased the levels of antioxidant enzymes while decreasing those of ROS in the hypothalamus.

The process of aging is associated with a redox imbalance and the accumulation of oxidative damage in many tissues, which is caused by an increase in ROS production and a decrease in antioxidant capacity (29). The hypothalamus is especially oxidatively challenged with increasing age and is vulnerable to oxidative stress (3). Since the hypothalamus plays a critical role in the central nervous system, age-related oxidative stress in this organ usually results in the loss of various physiological functions and the development of diseases such as age-related obesity and neurodegeneration. We found that long-term l-serine supplementation attenuated the decreases in the levels of antioxidants (GSH) and antioxidant enzymes (SOD) and the accumulation of ROS that are observed during aging. Based on these results, we believe that the functionality of the hypothalamus would be improved by long-term l-serine supplementation at an appropriate concentration in aging mice.

In age-related diseases, there is an antagonistic crosstalk between Sirt1 and NFκB. On the one hand, the inhibition of Sirt1 stimulates NFκB-induced inflammation directly through regulating the p65 subunit of the NFκB complex. On the other hand, NFκB inhibits Sirt1 activity mainly by affecting the production of factors such as ROS (30). Sirt1 plays a major role in the regulation of oxidative stress during aging. A reduction in Sirt1 expression with the progression of aging contributes to the upregulation of oxidative damage (31). Consequently, Sirt1-activating dietary supplements such as resveratrol, eicosapentaenoic acid, docosahexaenoic acid, and acetylshikonin show anti-aging effects (32, 33). Moreover, Sirt1 also plays a protective role against neuroinflammation in brain disease (33). Here, we found that l-serine supplementation at the concentration of 0.5% (w/v) restored the expression of Sirt1 after its age-related decrease. Thus, we suggest that the Sirt1 pathway may mediate the antioxidative and anti-inflammatory effects of l-serine in the hypothalamus of aging mice. Additionally, the activity of NFκB signaling was reported to increase in the hypothalamus with aging (34), suggesting that NFκB signaling is a promising target for preventing age-related inflammation. The inhibition of this pathway by caloric restriction and dietary nutrients such as polyunsaturated fatty acids delays aging and extends the lifespan of rodents (33, 35). Our results also indicate that l-serine attenuated the inflammatory response, which is usually associated with oxidative stress during aging, since the concentrations of inflammatory cytokines and the expression of NFκB decreased in the hypothalamus. These results indicated that NFκB pathway also plays an important role in mediating the preventive effects of l-serine against the increased inflammation in aged mice.

Unexpectedly, we found that long-term l-serine supplementation affected food intake and decreased age-related body weight gain. l-Serine has been used for the treatment of serine deficiency disorders and as a neuroprotective substance for decades (36). A phase I human clinical trial indicated that l-serine could be a generally safe supplement for patients with amyotrophic lateral sclerosis/motor neuron disease (37). However, we noticed that, in these reports, two patients showed a slightly decreased food intake. Supplementation with a high dose of l-serine for a long period might have some effects on food intake. The decreased expression of NPY and AGRP, which are highly conserved neuropeptides with orexigenic actions in the hypothalamus (38), could have contributed to the reduced food intake in aging mice treated with 0.5% l-serine. In addition, the increases in the expression of leptin receptor and the phosphorylation of STAT3, which is involved in the leptin signaling pathway in the hypothalamus, induced by serine would also affect food intake because of the anorectic effects of leptin. However, a high leptin level was found in aged mice; therefore, we suggest that leptin resistance may occur in these aged animals. The preventive effects of long-term l-serine supplementation on age-induced oxidative stress and age-related obesity observed herein suggest that l-serine supplementation might have the same effects as lifelong caloric restriction. Furthermore, l-serine supplementation in aging mice activated *Sirt1*, which is defined as a “longevity gene,” suggesting a life-extending effect of serine. Interestingly, researchers found that in the village of Ogimi, which is known as the “Village of Longevity” in Japan, the traditional food items of residents are rich in l-serine (25). Further studies are required to provide more convincing evidence of the life-extending effect of l-serine.

In conclusion, our results suggest that long-term l-serine supplementation at an appropriate concentration attenuates age-related oxidative stress and the inflammatory response in the hypothalamus of aging mice. The Sirt1 and NFκB pathways may mediate these effects of l-serine. However, the molecular mechanisms through which l-serine decreases the expression of NFκB while rescuing that of Sirt1 need to be further elucidated. Additionally, we unexpectedly found that l-serine reduced food intake and age-related body weight gain. l-Serine may exert these effects by regulating the leptin pathway and the orexigenic neuropeptides NPY and AGRP.

## Author contributions

XZ, LH, and HZ conducted the experiment. XZ and LH collected and analyzed the data. XW and YY helped with the discussion. XZ and XW designed the experiment and wrote the manuscript. XW and YY revised the manuscript.

### Conflict of interest statement

The authors declare that the research was conducted in the absence of any commercial or financial relationships that could be construed as a potential conflict of interest.
