# Supplementary material for: Long-Term l-Serine Administration Reduces Food Intake and Improves Oxidative Stress and Sirt1/NFκB Signaling in the Hypothalamus of Aging Mice
Source: Front Endocrinol (Lausanne). 2018 Aug 23;9:476. doi: 10.3389/fendo.2018.00476 (PMC6115525; doi:10.3389/fendo.2018.00476)
Supplement: Supplementary file 1 [file Table_1.DOCX]

**Supplementary Table 1** Primer sequences

| Gene | 5’-3’ Primer sequence |
| --- | --- |
| LepRb | F: GAAGATGATGGAATGAAGTG |
|  | R: GAGCAGTAGGACACAAGAGG |
| NPY | F: TCACAGAGGCACCCAGAG |
|  | R: TGTCGCAGAGCGGAGTAG |
| AGRP | F: TACCTTGCTGCGACCCG |
|  | R: TGCCCAAACAACATCCATT |
| β-actin | F: TGTCCACCTTCCAGCAGATGT |
|  | R: AGCTCAGTAACAGTCCGCCTAGA |
